# Blood Pressure in Healthy Humans Is Regulated by Neuronal NO Synthase

**DOI:** 10.1161/HYPERTENSIONAHA.116.08792

**Published:** 2017-04-12

**Authors:** Husain Shabeeh, Sitara Khan, Benyu Jiang, Sally Brett, Narbeh Melikian, Barbara Casadei, Philip J. Chowienczyk, Ajay M. Shah

**Affiliations:** From the King’s College London British Heart Foundation Centre, Cardiovascular Division, United Kingdom (H.S., S.K., B.J., S.B., N.M., P.J.C., A.M.S.); and Department of Cardiovascular Medicine, University of Oxford, United Kingdom (B.C.).

**Keywords:** blood pressure, brain, humans, nitric oxide, stroke

## Abstract

NO is physiologically generated by endothelial and neuronal NO synthase (nNOS) isoforms. Although nNOS was first identified in brain, it is expressed in other tissues, including perivascular nerves, cardiac and skeletal muscle. Increasing experimental evidence suggests that nNOS has important effects on cardiovascular function, but its composite effects on systemic hemodynamics in humans are unknown. We undertook the first human study to assess the physiological effects of systemic nNOS inhibition on basal hemodynamics. Seventeen healthy normotensive men aged 24±4 years received acute intravenous infusions of an nNOS-selective inhibitor, S-methyl-l-thiocitrulline, and placebo on separate occasions. An initial dose-escalation study showed that S-methyl-l-thiocitrulline (0.1–3.0 µmol/kg) induced dose-dependent changes in systemic hemodynamics. The highest dose of S-methyl-l-thiocitrulline (3.0 µmol/kg over 10 minutes) significantly increased systemic vascular resistance (+42±6%) and diastolic blood pressure (67±1 to 77±3 mm Hg) when compared with placebo (both *P*<0.01). There were significant decreases in heart rate (60±4 to 51±3 bpm; *P*<0.01) and left ventricular stroke volume (59±6 to 51±6 mL; *P*<0.01) but ejection fraction was unaltered. S-methyl-l-thiocitrulline had no effect on radial artery flow-mediated dilatation, an index of endothelial NOS activity. These results suggest that nNOS-derived NO has an important role in the physiological regulation of basal systemic vascular resistance and blood pressure in healthy humans.

**See Editorial Commentary, pp 778–779**

NO is a crucial physiological signaling molecule in diverse organ systems. NO is synthesized from L-arginine and molecular oxygen by a family of 3 NO synthases (NOSs): endothelial NOS (eNOS), neuronal NOS (nNOS), and inducible NOS, which have distinct roles and functions.^1^ eNOS and nNOS are constitutively expressed isoforms named after the cell type in which they were first identified but are also present in other tissues. eNOS is particularly important in the cardiovascular system where it is involved in multiple homeostatic processes, including the endothelium-dependent regulation of vascular tone and blood flow, inhibition of platelet aggregation and adhesion, modulation of cardiac contraction, inhibition of vascular smooth muscle proliferation, and promotion of angiogenesis.^1^ nNOS is found in the central nervous system, peripheral nerves (termed nitrergic nerves), and many other tissues, including cardiac and skeletal muscle.^2^

Although eNOS is well established to be of significant importance in cardiovascular physiology, it is increasingly evident from animal studies that nNOS is also involved in the regulation of cardiovascular function and exerts effects that in general are distinct from those of eNOS.^1,3^ Several studies using nNOS-selective inhibitors or nNOS-deficient mice suggested that nNOS-derived NO exerts central effects on blood pressure (BP) by regulating sympathetic outflow, although some studies in nNOS knockout mice reported no effect on BP.^3,4^ nNOS may also influence BP by modulating renal renin release and fluid balance.^5^ nNOS in nitrergic nerves modulates vessel tone at a local level in several vascular beds, which could potentially impact on BP.^3^ Other experimental studies indicate an important role for nNOS in regulating changes in heart rate (HR) mediated by baroreflex responses.^6^ Finally, nNOS regulates cardiac excitation–contraction coupling in mice, in particular influencing myocardial relaxation and the response to β-adrenergic stimulation.^7^

These studies suggest that nNOS may have an important role in regulating the cardiovascular system, but this possibility has not been directly investigated in humans. We previously reported studies in which an nNOS-selective inhibitor, S-methyl-l-thiocitrulline (SMTC), was infused locally into the brachial artery or coronary artery of healthy humans. Local infusion of SMTC reduced basal blood flow both in the forearm and coronary circulations without affecting the eNOS-mediated vasodilator response to acetylcholine, substance P, or increased shear stress.^8,9^ Additional studies showed that local infusion of SMTC inhibited mental stress–induced increases in forearm blood flow but had no effect on pacing-induced increases in coronary blood flow.^8,10^ These findings suggest that nNOS-derived NO contributes to the tonic regulation of human microvascular tone, at least in the coronary and forearm skeletal muscle vascular beds. Previous studies in which the nonselective NOS inhibitor, *N*^G^-monomethyl-l-arginine (L-NMMA), was infused systemically in humans reported a transient pressor response.^11–13^ However, the potential contribution of nNOS-derived NO cannot be ascertained from these studies because L-NMMA inhibits all NOS isoforms. The aim of this study was to undertake the first direct investigation in healthy humans of the hemodynamic effects of systemic nNOS inhibition.

## Methods

The study conformed to the standards set by the Declaration of Helsinki and received local Research Ethics Committee and Research Governance approval. The study protocol was submitted to the UK Medicines and Healthcare products Regulatory Agency and was not classed as a Clinical Trial of an Investigational Medicinal Product (CTIMP). However, given that SMTC had not previously been administered systemically (intravenously) to humans, studies were initiated with a no-effect dose, and appropriate safety checks were performed. All participants provided written informed consent. We studied 17 lean healthy male subjects aged 24±4 years, who were recruited by advertisement. No subject had a history of smoking, recreational drug use, excessive alcohol intake, or use of over the counter medicines over the previous 3 months. All underwent clinical screening, ECG, and blood hematology and biochemistry profiles to exclude hyperlipidemia, diabetes mellitus, hypertension, renal, and liver disease. Participants were instructed to abstain from caffeine and alcohol for at least 12 hours before studies.

### SMTC Dosing and Protocol

Our previous studies with local intra-arterial administration showed that SMTC is ≈10-fold more potent than L-NMMA at increasing basal forearm vascular resistance.^8^ Previous studies in which L-NMMA was administered systemically (intravenously) reported a 30% to 40% increase in systemic vascular resistance (SVR) with doses of 12 to 24 µmol/kg (bolus over 5 minutes).^12,13^ We therefore chose a maximum dose of SMTC that was ≈10-fold lower, that is, 3 µmol/kg. We calculated the total SMTC dose that we had previously used in local intra-arterial infusion studies^8–10^ (0.1 µmol/kg) and used this as a starting point for an ascending dose study. All SMTC doses were administered over 10 minutes into a large antecubital vein.

Each SMTC dose or placebo was administered on a separate occasion at least a week apart. In the first 3 subjects, we assessed escalating single intravenous doses of SMTC (0.1, 0.3, 1.0, and 3.0 µmol/kg) and 1 placebo dose (saline vehicle) randomized in relation to visits in which rising doses of SMTC were administered. The next 6 subjects received SMTC (1.0 and 3.0 µmol/kg) and a randomized placebo dose. The final 8 subjects received SMTC (3.0 µmol/kg) and placebo in random order. Therefore, 9 subjects received SMTC 1.0 and 3.0 µmol/kg, and all 17 subjects received SMTC 3.0 µmol/kg. The individuals undertaking hemodynamic and other measurements were blinded to the treatment (except in the case of the first 3 subjects for safety reasons). Participants returned for clinical review, and for repeat hematology, renal and liver profiles 3 to 5 days after each dose of SMTC.

### Hemodynamic Assessment

All studies were undertaken in a quiet temperature-controlled vascular laboratory after at least 30 minutes of supine rest. A 16-gauge cannula was inserted into a large antecubital vein for infusions. The ECG was continuously monitored. HR and BP were measured using a standard oscillometric method. Left ventricular (LV) stroke volume was measured by 3-dimensional echocardiography in the last 8 subjects. SVR was calculated as mean arterial pressure (MAP) divided by cardiac output (CO). Measurements of hemodynamic parameters were made every 5 minutes for 15 minutes after completion of SMTC infusion and then every 30 minutes for 3 hours.

### Echocardiography

Transthoracic echocardiography was performed according to American Society of Echocardiography guidelines,^14^ using a Vivid 7 system (GE Medical, United Kingdom) with 2.5 MHz matrix array and stand-alone transducers. For 3-dimensional (3D) echocardiography, full-volume data sets were obtained from the apical 4-chamber window over 4 consecutive cardiac cycles and analyzed offline. LV stroke volume and ejection fraction were calculated offline using semiautomated border tracking in 3 orthogonal views. All echocardiography acquisition and data analyses were performed by an experienced operator who was blinded to interventions.

### Flow-Mediated Dilatation

To assess whether SMTC might be affecting eNOS-dependent responses, we quantified radial artery flow-mediated dilatation (FMD) as described previously^9^ in the final 8 subjects who received SMTC (3.0 µmol/kg) and placebo. In brief, images of the radial artery were acquired using high-resolution B-mode ultrasound with a 7-MHz linear array transducer (Acuson Aspen Advanced Imagegate). A BP cuff 5 to 10 cm distal to the transducer was inflated to at least 50 mm Hg above systolic pressure for 5 minutes, followed by release to induce reactive hyperemia. FMD was measured at baseline before any infusions and then again immediately after the completion of the SMTC infusion (which was found to be the time point of maximal hemodynamic response). All scanning and FMD analysis was performed by an experienced operator who was blinded to interventions.

### Statistical Analysis

Data are presented as mean± SEM. Comparisons were made by repeated measures ANOVA or 2-tailed paired *t* test as appropriate. *P* value <0.05 was considered significant.

## Results

The administration of SMTC did not result in any significant clinical or other adverse effects. SMTC doses of 0.1 and 0.3 µmol/kg, which were tested in 3 subjects, seemed to have no effect on HR or BP. This was expected on the basis of previous studies with local intra-arterial infusion of SMTC, where no effect on BP was found.^8,9^

### Dose-Dependent Effect of SMTC on BP

**Figure 1.**
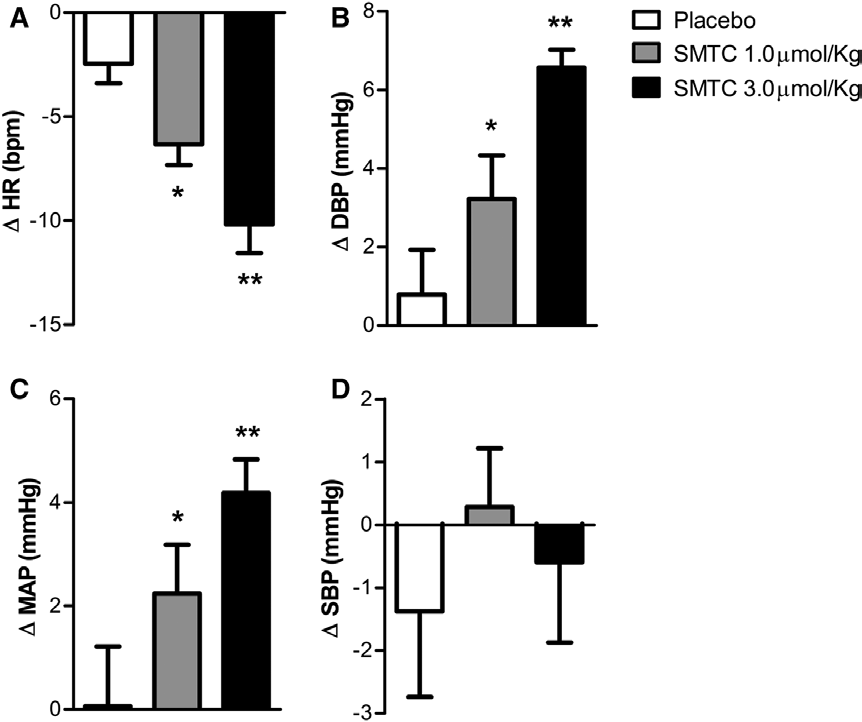
Change from baseline of heart rate and blood pressure immediately after infusion of S-methyl-l-thiocitrulline (SMTC; 1.0 µmol/kg) and SMTC (3.0 µmol/kg) and saline vehicle placebo over 10 min. **A**, Heart rate (ΔHR); (**B**) diastolic blood pressure (ΔDBP); (**C**) mean arterial pressure (ΔMAP); and (**D**) systolic blood pressure (ΔSBP). *P<0.05 compared with placebo; **P<0.01 compared with placebo.

**Figure 2.**
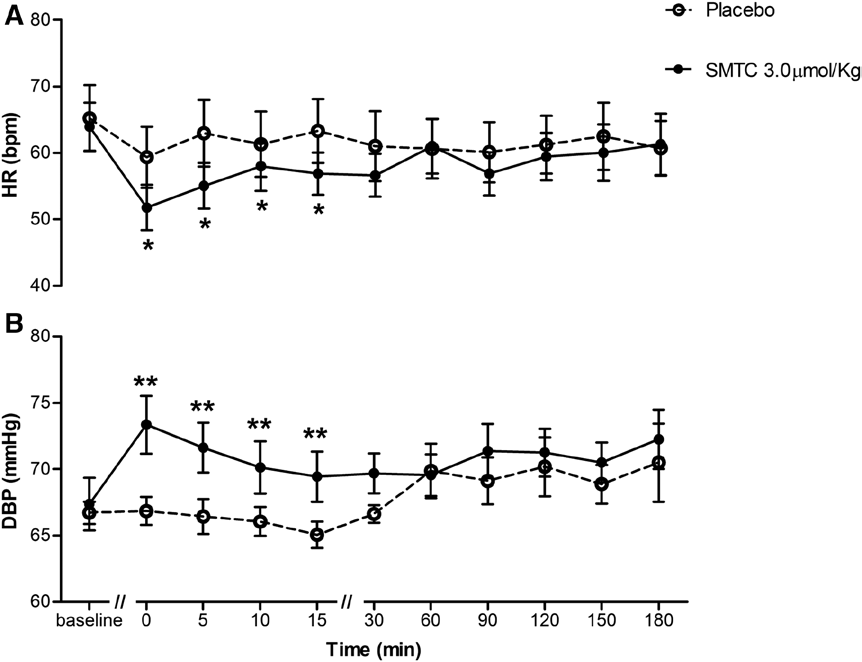
Time course of hemodynamic (heart rate [HR] and diastolic blood pressure [DBP]) response to S-methyl-l-thiocitrulline (SMTC; 3.0 µmol/kg). **A**, HR and (**B**) DBP. Time is measured after infusion of SMTC over 10 min. *P<0.05, **P<0.01 compared with placebo for the analysis of variance for repeated measures over the time period from 0 to 15 min after completion of infusion of SMTC.

SMTC (1.0 and 3.0 µmol/kg) had dose-dependent hemodynamic effects when compared with placebo infusion. It significantly increased diastolic BP and MAP, whereas HR was significantly decreased (n=9; each *P*<0.01; Figure 1; Table). There was no significant effect on systolic BP. The maximal response to SMTC was observed 10 minutes after initiation of infusion, and the changes in HR and BP gradually returned to baseline over the next 30 to 60 minutes. The time course of changes in HR and diastolic BP is illustrated in Figure 2.

**Table.**
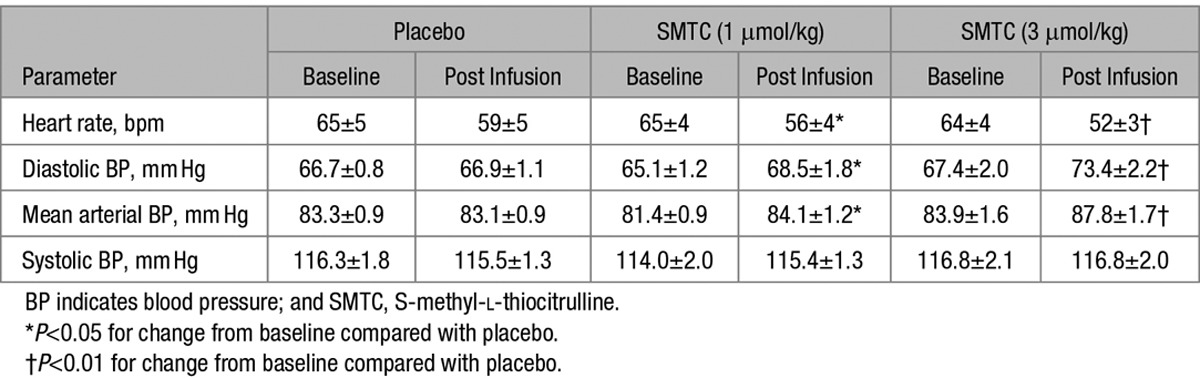
Heart Rate and BP Before and After a 10 Minutes Infusion of SMTC and Placebo

### Effect of SMTC (3.0 µmol/kg) on Hemodynamics and Cardiac Function

**Figure 3.**
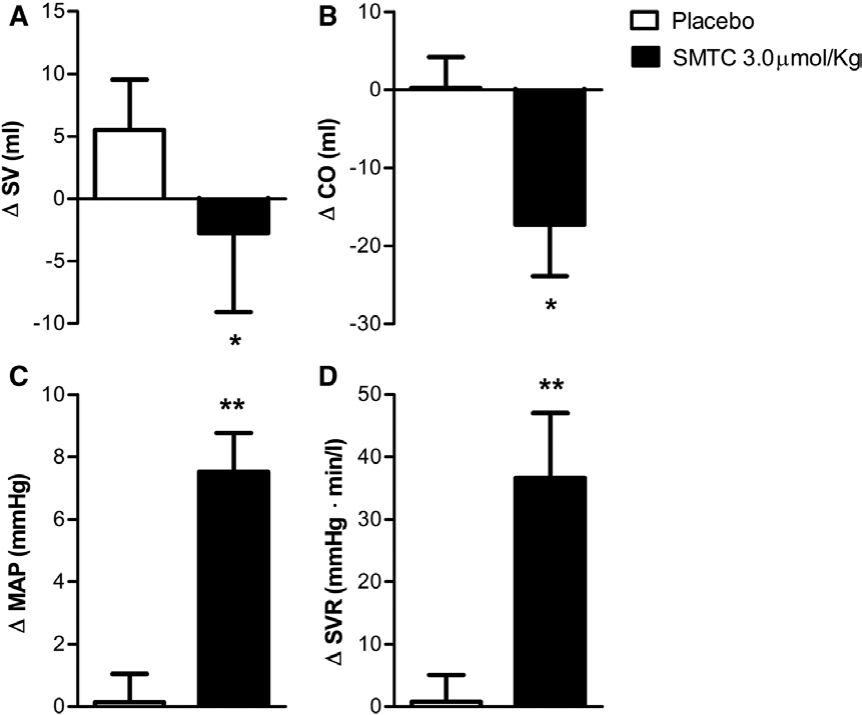
Change from baseline of (**A**) stroke volume (SV), (**B**) cardiac output (CO), (**C**) mean arterial blood pressure (MAP), and (**D**) systemic vascular resistance (SVR) immediately after infusion of S-methyl-l-thiocitrulline (SMTC; 3.0 µmol/kg) and saline vehicle placebo over 10 min. *P<0.05 compared with placebo; **P<0.01 compared with placebo.

All 17 study participants received the highest dose of SMTC, while in 8 subjects we also performed 3D echocardiography to assess cardiac function. In these subjects, changes in HR and BP were similar to those in the first 9 subjects, with diastolic BP increasing by 10±2 mm Hg (*P*<0.001) and MAP by 7±2 mm Hg (*P*<0.01), whereas HR was reduced by 6±1 bpm (*P*<0.01). The SMTC-induced changes in echocardiographic measures of cardiac function are shown in Figure 3. There was a significant decrease in LV stroke volume (−14±3%; *P*<0.01), related to an increase in LV end-systolic volume with no change in LV end-diastolic volume. The increase in MAP and the decrease in CO were associated with an increase in SVR of 42±6% (*P*<0.001) when compared with placebo. Ejection fraction and LV stroke work, however, were not altered by SMTC (data not shown).

### Effect of SMTC (3.0 µmol/kg) on FMD

**Figure 4.**
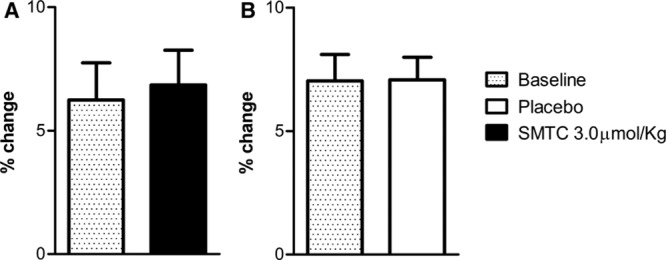
Flow-mediated dilation (FMD) before and 10 min after infusion of saline vehicle placebo and S-methyl-l-thiocitrulline (SMTC; 3.0 µmol/kg).

In 8 subjects, we compared the effects of SMTC (3.0 µmol/kg) or placebo on FMD, an index of eNOS-dependent vasodilatation.^1^ Neither SMTC nor placebo infusion had any significant effect on baseline radial artery diameter or on FMD (Figure 4).

### Serum SMTC Concentration

Serum concentrations of SMTC were undetectable at baseline. However, 20 minutes after administration of a dose of 3.0 µmol/kg, shortly after the time of maximal effect on BP, the mean serum concentration was 80.5±10.8 ng/mL (≈0.29 µmol/L) and declined to 22.8±3.9 ng/mL after 1 hour (n=7).

## Discussion

Our previous first-in-human studies with the nNOS-selective inhibitor SMTC indicated that nNOS has a major role in the basal regulation of microvascular tone in the forearm and coronary circulations, whereas eNOS mediates relaxant responses to pharmacological and shear stress stimuli.^8,9^ We have now undertaken the first human studies to assess the integrated hemodynamic effects of systemic nNOS-selective inhibition with SMTC in healthy subjects. We found that SMTC induces a significant increase in SVR and BP and a reduction in HR and stroke volume. The highest dose of SMTC that was studied (3 µmol/kg) increased SVR by over 40%, whereas LV stroke volume was reduced by about 15%. This dose of SMTC was expected to be nNOS-selective based on our previous human studies with local infusion, in which the archetypal eNOS-mediated responses of FMD or acetylcholine-induced vasodilation were unaffected by SMTC.^8,9^ Indeed, we confirmed the lack of effect of systemic SMTC on FMD in this study, consistent with a previous study in which local brachial artery infusion of SMTC reduced forearm blood flow but had no effect on radial artery diameter or radial artery FMD.^9^ Furthermore, the measured serum concentration of SMTC at the time of peak hemodynamic effect was ≈0.29 µmol/L, which is significantly below the level at which any significant eNOS inhibition might occur.^15,16^ Previous studies demonstrated the selectivity of SMTC for nNOS over eNOS, not only in rodent tissues but also in assays with the human enzymes.^15^

The magnitude of effect of systemic SMTC on BP and SVR was broadly similar to results previously observed with the use of systemic nonselective NOS inhibition with L-NMMA.^11–13^ However, our maximum dose of SMTC was ≤8× smaller than the dose of L-NMMA that caused similar hemodynamic effects,^13^ an approximate dose ratio which has been shown to be nNOS selective in forearm studies.^8^ Taken together, these considerations imply that a major component of the systemic hemodynamic effects of L-NMMA in healthy men may in fact be related to the inhibition of nNOS rather than eNOS. Our findings are also consistent with previous animal studies in which different nNOS-selective inhibitors increased BP in rats in vivo.^5,16^ However, our findings with regard to the physiological regulation of BP by nNOS rather than eNOS differ from those inferred from genetically modified murine models in which eNOS but not nNOS knockout mice are hypertensive.^1,17^ Interestingly, a human study that used systemic infusion of a different nonselective NOS inhibitor, N^G^-nitro-L-arginine methyl ester (L-NAME), reported a much larger increase in BP than that observed with L-NMMA.^18^ In this study, we did not assess the maximal response to SMTC (because we wished to avoid the possibility of concurrent eNOS inhibition). Therefore, it is possible that the maximal physiological nNOS-dependent effect on BP could be higher than was observed in the current study.

Among the first to use systemic L-NMMA in humans were Haynes et al,^12^ who found that L-NMMA infused systemically at a dose of 3 mg/kg over 5 minutes produced an increase in diastolic BP and MAP of ≈7 mm Hg, with a decrease in HR of 14 bpm, when compared with placebo. No significant increase in SBP was observed in their study. Using a noninvasive bioimpedance method, they found that cardiac index decreased by 25±4% and SVR increased by 46±12%. Stamler et al^11^ used invasive measurement of BP and CO (Fick method) during systemic infusion of L-NMMA (3 mg/kg over 3 minutes) and observed a 15% increase in MAP and a 63% increase in SVR. These previous results support the notion that NO influences SVR through the local regulation of vascular tone and thereby alters BP. This study suggests that a substantial component of the above effects may be attributable to inhibition of nNOS rather than eNOS. On the basis of our previous studies of the effects of local intra-arterial infusion of SMTC on microvascular tone,^8,9^ a major mechanism through which systemic SMTC acts to raise BP is likely to be the inhibition of nNOS in the microvasculature. We have suggested that the local vasodilator action of nNOS may be the result of NO release from perivascular nitrergic nerves, as in published animal studies.^3^ It has also been suggested that nNOS may release hydrogen peroxide when the levels of its substrate L-arginine are nonsaturating^19^; because hydrogen peroxide is a vasodilator in the microvasculature, this could be another underlying mechanism. In addition to local microvascular effects, a significant component of the action of nNOS on BP may involve central effects on sympathetic outflow.^3,4^ In previous human studies using L-NAME, it was suggested that 40% of the pressor effect may be central,^18^ consistent with other studies suggesting that NO is involved in the central regulation of sympathetic outflow in humans.^20^ Finally, animal studies suggest that an effect of nNOS-derived NO on renal renin release could also be important in BP regulation.^5^ Additional studies will be required to define the relative contributions of these different mechanisms in the pressor effects of SMTC.

The decrease in HR and CO observed during SMTC infusion could have several explanations. The most straightforward reason for a decrease in CO is the increase in SVR (ie, increased afterload). It is also possible that the increase in BP initiates a baroreceptor reflex resulting in withdrawal of sympathetic efferent activity and augmentation of vagal activity, causing a decrease in HR and CO.^21^ Stamler et al^11^ suggested that the effects of L-NMMA on HR and CO could not be explained solely by the increase in BP because similar changes in BP induced by the α1-adrenergic receptor agonist, phenylephrine, did not cause an equivalent decrease in CO. These authors, therefore, postulated that there might also be direct cardiac effects or specific effects of NO on sympathetic outflow. On the other hand, Hansen et al^22^ found that systemic infusion of either L-NMMA or phenylephrine caused similar increases in BP and similar reduction in HR and sympathetic nerve activity, suggesting that the effects of L-NMMA on HR and CO might be secondary to changes in BP. A direct cardiac effect of SMTC might be possible; however, nNOS-derived NO is known to have a negative inotropic effect in vivo in the mouse and in isolated cardiomyocytes.^7,23^ We found no changes in ejection fraction, suggesting a lack of major impact of SMTC on basal LV systolic function. However, analysis of contractile function by higher-fidelity methods (eg, pressure-volume analysis) would be required to make definitive conclusions. In addition, previous animal work suggests that nNOS-derived NO specifically affects diastolic function and β-adrenergic inotropic responsiveness.^24^ Additional studies are, therefore, required to assess whether nNOS-derived NO has similar effects in the human heart.

The results of this study may have potential clinical implications. The hallmark of essential hypertension is an increase in peripheral vascular resistance. However, eNOS-stimulated responses have been shown to be relatively preserved in patients with essential hypertension when compared with patients with other risk factors for cardiovascular disease, such as diabetes mellitus and hypercholesterolemia.^25,26^ Our findings provide a potential explanation for this discrepancy and raise the important question of whether nNOS dysfunction is an important contributor to hypertension. In this regard, we have recently shown that mental stress–induced vasodilatation in the forearm, a response shown to be mediated at least in part by local nNOS-derived NO, is impaired in many patients with hypertension.^27^ Another clinical scenario in which the impact of nNOS dysfunction merits investigation is chronic heart failure, which is characterized by a significant increase in SVR. Patients with heart failure have a decreased vasomotor response to intracoronary L-NMMA, suggesting that basal release of NO in the coronary circulation is reduced in these patients,^28^ and this might involve a decrease in nNOS-derived NO based on our previous work in the human coronary circulation.^9^ Furthermore, patients with chronic heart failure also had enhanced inotropic responses to β-adrenergic agonists after intracoronary L-NMMA.^29^

### Study Limitations

This study was performed in healthy young men and should be replicated in other groups, including subjects with cardiovascular risk factors. No definitive conclusions can be made about the direct effects of nNOS-derived NO on cardiac function or autonomic function based on this study. Additional studies designed to specifically address these aspects and to define the relative impact of central versus peripheral nNOS inhibition would be valuable. Evidence that SMTC exerts specific effects solely on NOS was based on the lack of effect on FMD, a prototypic eNOS-mediated response. Possible effects of SMTC on the hyperemic blood flow stimulus to FMD could have influenced the FMD response but would have tended to bias toward a reduction in FMD. Should other specific inhibitors of nNOS become available for human use, studies should be undertaken with such agents to confirm the current data.

### Perspectives

Although the physiological effects of nNOS have been extensively studied in animals, no previous human studies have addressed the impact of nNOS on the regulation of systemic hemodynamics and BP. Here, we report the first human studies to investigate the integrated hemodynamic effects of nNOS inhibition. Our results indicate that nNOS has an important role in the physiological regulation of SVR and BP in healthy humans. The precise site(s) of nNOS action through which these effects are mediated require further investigation. The current work provides a foundation for future studies to investigate whether nNOS dysfunction is implicated in disease states in which systemic hemodynamics are altered.

## Acknowledgments

We thank Professor Tim Mant, Honorary Professor in Translational Medicine, King’s College London for providing advice on dose escalation and independent review of the protocol.

## Sources of Funding

The British Heart Foundation (RE/13/2/30182; FS/09/062/27958) and the Department of Health via a National Institute for Health Research (NIHR) Biomedical Research Center and Clinical Research Facility award to Guy’s and St Thomas’ National Health Service Foundation Trust in partnership with King’s College London.

## Disclosures

None.

## Novelty and Significance

### What Is New?

- A neuronal NO synthase (nNOS)–selective inhibitor, S-methyl-L-thiocitrulline, was tested systemically for the first time in humans.
- Intravenous S-methyl-L-thiocitrulline infusion elevates blood pressure in healthy men.

### What Is Relevant?

- nNOS has an important role in the physiological regulation of human systemic vascular resistance and blood pressure.
- The site(s) of nNOS action involved in these effects require further study.

### Summary

This is the first study to identify a physiological role for nNOS in regulating human blood pressure. S-methyl-L-thiocitrulline may be a useful tool to investigate the impact of nNOS dysfunction in disease.
